# Case of Aneurism of the Aorta Simulating Phthisis

**Published:** 1852-01

**Authors:** W. B. McCready

**Affiliations:** Physician to Bellevue Hospital


ARTICEL II.

*Case of Aneurism of the Aorta simulating Phthisis.* By W. B. McCready, M. D,, Physician to Bellevue Hospital.

Andrew Quinn, laborer, born in Ireland, was admitted into Bellevue Hospital on the 27th of September, 1851. The patient was about 45 years of age, with dark hair and eyes, and much emaciated. His mother and a brother had died of phthisis. Until two years ago, he had always enjoyed good health ; at this time, he was troubled with some slight pain in the lower part of the chest, shooting toward the right side, and afterwards felt upon the left. Five months ago he was attacked with a dry, hacking cough ; this was soon afier attended with slight expectoration, and three months ago, the sputa began to be tinged with blood. About the same time he commenced to feel warmer towards evening, and to be troubled with night sweats. Since then he has been gradually emaciating. On the 22d September, after a severe fit of coughing, he raised about a gill of thick, dark-colored blood, mixed with air-bubbles. He has since continued daily to spit blood.

When admitted, the skin was cool and moist; the pulse 80, small and weak; the respiration 25 in a minute, and the tongue clean. The voice was feeble but not hoarse, the cough soft, and there was no pain in the chest.

On physical examination the Physician in attendance found marked flatness, with tubular respiration, under the right clavicle, under the left, the expiratory murmur was somewhat prolonged. Posteriorly, there was some flatness in the superior scapular regions, with, on the right side, marked prolongation of expiratory murmur.

The patient continued to expectorate mixed blood and mucus in considerable qnantity, filling a pint cup in twenty-four hours. On the 3d of October, after a severe paroxysm of coughing, he raised a pint and a half of pure, bright-colored blood. After this, he remained exceedingly feeble. He continued daily to raise blood, gradually sinking, till on the 16th of October, on making the evening visit, the house physician found him with the blood flowing in a stream from the nose and mouth. He died at seven P. M.

*Post mortem examination*, twenty hours after death. Body exceedingly emaciated. On opening the chest no trace of tubercle was found in the lungs. An aneurism, of the size of a large orange, flattened in its antero-posterior diameter, lay directly behind the heart, occupying the posterior part of the aorta, just below the arch. On removing the lungs, together with the heart and descending aorta, it was found that the posterior wall of the aneurism was formed by a coagulum, adhering to the bodies of the vertebræ, which were denuded and carious. At the upper and left part of the sac, a large opening, admitting two fingers, communicated with the upper part of the lower globe of the left lung, near its root. Here the blood, partly forcing its way into the lung substance, partly pushing the lung before it, had formed a large cavity, about two inches and a half in diameter, almost completely filled with a coagulum. Three large bronchi (of the third division) had been partially denuded, and their walls very much thinned. In these two apertures, that would readily admit a small sized probe, with a number of others apparently of capilliary size existed. Through these openings, air freely bubbled when the lungs were inflated under water. In the neighborhood of the affected part, the lung was inflamed. Posteriorly, and to the left, where the walls of the aneurismal sac were deficient, the edges of the opening were well defined, thick and rounded. The lining membrane of the Aorta, as far as traced, was thickened and covered with patches of atheroma. The heart itself was healthy. As the examination was made in haste, the friends being in waiting for the body, the other organs were not examined.

The patient was passed over to me when I took possession of the Ward, on the 1st of the month, as a case of phthisis. Up to the third instant, I had no opportunity of making any physical examination, and after the alarming hemorrhage had occurred on that day, I did not deem it advisable to fatigue or disturb him. If it had been made it is not probable that the real disease would have been detected. The hereditary predisposition, the history of the case, the great emaciation, the very look of the patient pointed to phthisis alone.

If accident had directed the attention of the auscultator to the spinal column, perhaps a correct diagnosis might have been formed, though this is far from being certain.—*Ibid.*

